# Supplementary material for: Facile Synthesis of Black Phosphorus Nanosheet@NaReF4 Nanocomposites for Potential Bioimaging
Source: Nanomaterials (Basel). 2022 Sep 27;12(19):3383. doi: 10.3390/nano12193383 (PMC9565442; doi:10.3390/nano12193383)
Supplement: Supplementary file 1 [file nanomaterials-12-03383-s001.zip › nanomaterials-1856090-SM.pdf]

Supporting Information

Facile Synthesis of Black Phosphorus Nanosheet@$NaReF_4$ Nanocomposites for Potential Bioimaging

Dongya Wang [1], Jingcan Qin [1], Chuan Zhang [2,*] and Yuehua Li [1,*]

[1] Department of Radiology, Shanghai Jiao Tong University Affiliated Sixth People's Hospital, Shanghai Jiao Tong University School of Medicine, 600 Yi Shan Road, Shanghai 200233, China

[2] School of Chemistry and Chemical Engineering, Frontiers Science Center for Transformative Molecules, and State Key Laboratory of Metal Matrix Composites, Shanghai Jiao Tong University, 800 Dongchuan Road, Shanghai 200240, China;

* Correspondence: liyuehua77@sjtu.edu.cn (Y. L); chuanzhang@sjtu.edu.cn (C. Z)

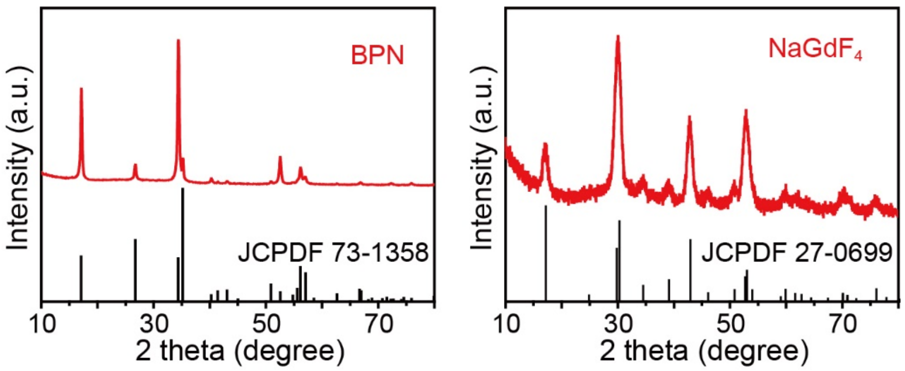

Figure S1. Powder XRD patterns of as-prepared BPN and $NaGdF_4$. The diffraction pattern at the bottom is the literature reference for black phospohrus (Joint Committee on Powder Diffraction Standards file number 73-1358) and hexagonal $NaGdF_4$ crystal (Joint Committee on Powder Diffraction Standards file number 27-0699).

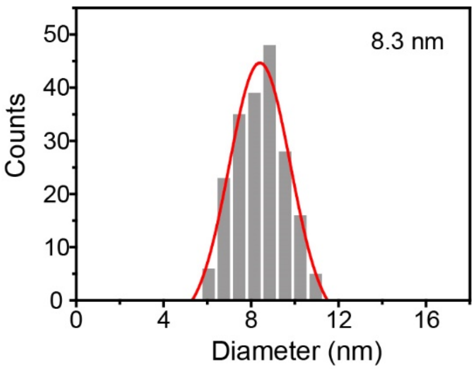

Figure S2. Size distribution of the oleic acid capped $NaGdF_4$ nanoparticles.

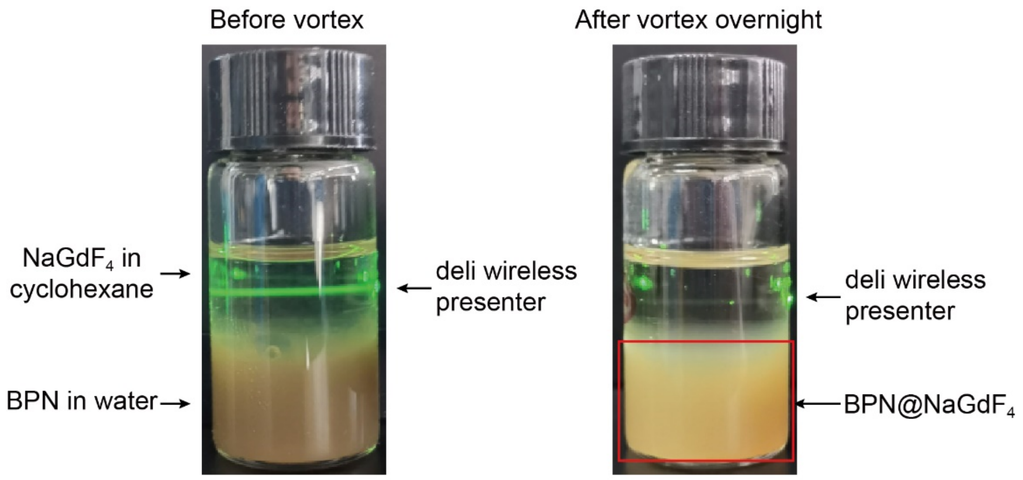

Figure S3. Pictures of reaction bottle containing $NaGdF_4$ and BPN before and after reaction. The green light beam is originated from Tyndall effect when the solution contained nanoparticles is irradiated by a laser. The weaker light intensity after reaction indicates that the amount of nanoparticles is decreased.

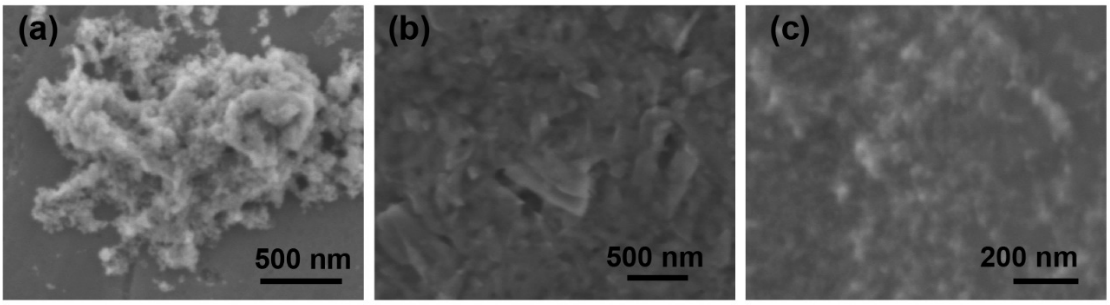

Figure S4. SEM images of the $NaGdF_4$ (a), BPN (b) and BPN@$NaGdF_4$ nanocomposites (c). Because of the poor conductivity of BPN and $NaGdF_4$, SEM images are not clear enough.

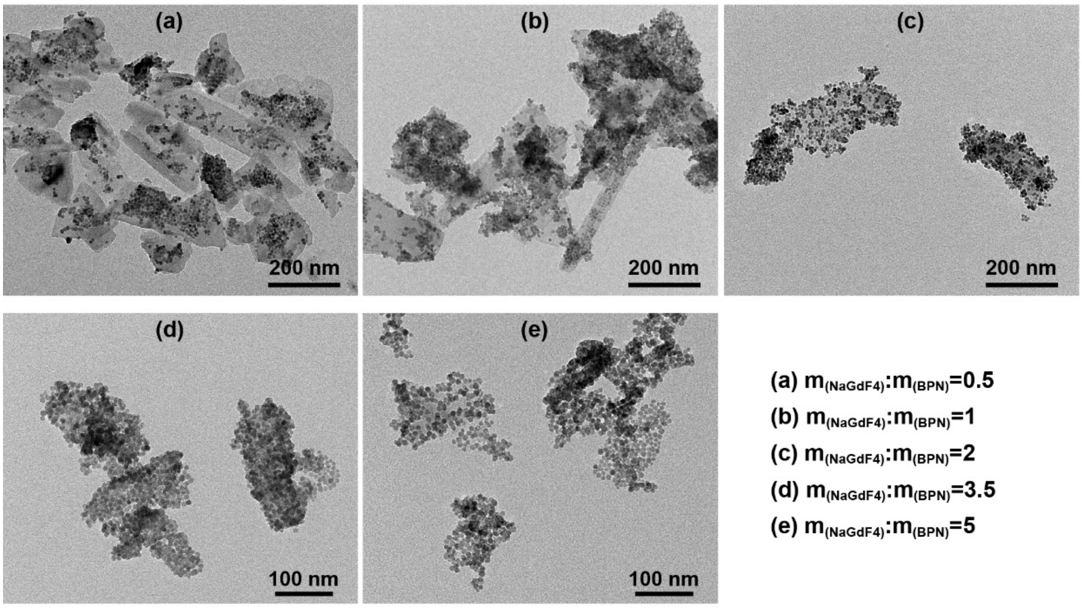

Figure S5. TEM images of BPN@$NaGdF_4$ for different feeding ratios of $m_{(NaGdF4)}$:$m_{(BPN)}$. As the images show, the number of $NaGdF_4$ nanoparticle on BPN keeps constant when the feeding ratio is higher than 2.

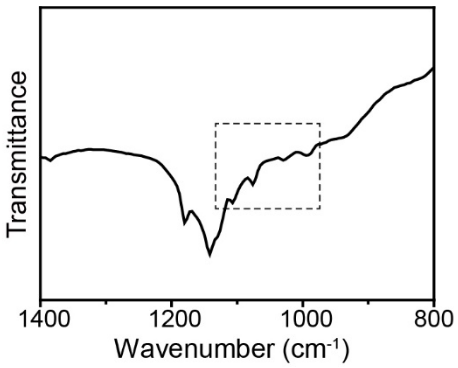

Figure S6. FTIR spectrum of as-synthesized BPN. The peaks in 1115-975 cm^{-1} range are from phosphate.

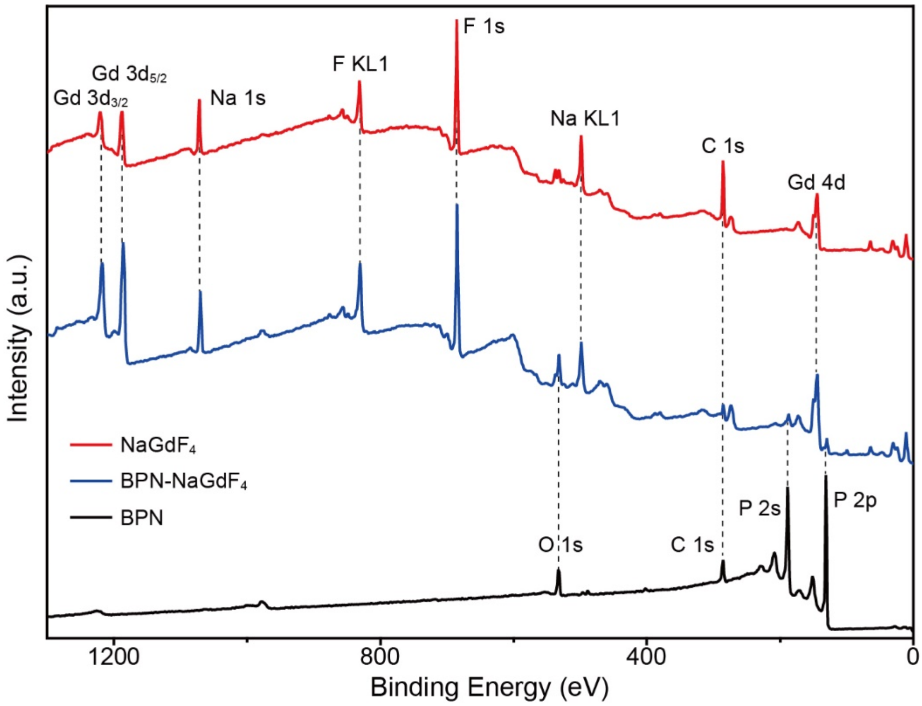

Figure S7. XPS survey spectra of the BPN, $NaGdF_4$ and BPN@$NaGdF_4$ nanocomposites. For the BPN@$NaGdF_4$ sample, the XPS peaks of all the elements including Na, Gd, F and P appears meaning the integration of BPN and $NaGdF_4$.

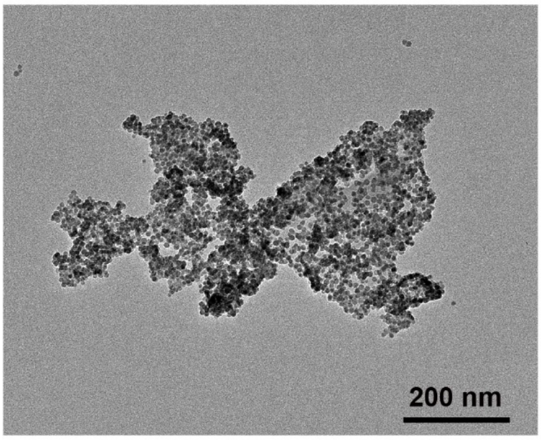

Figure S8. TEM image of BPN@$NaGdF_4$ nanocomposites after rigorously stirring overnight.

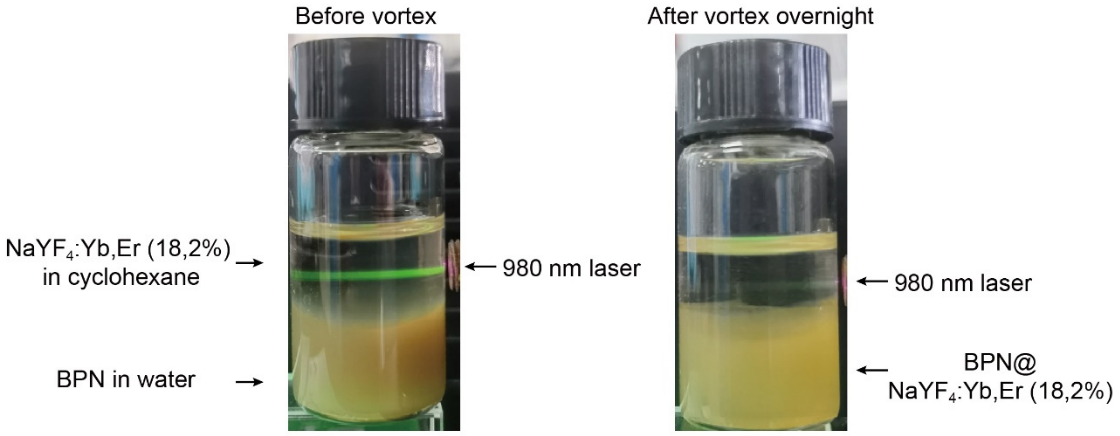

Figure S9. Pictures of reaction bottle containing $NaYF_4$:Yb,Er (18,2%) and BPN before and after reaction. The green light beam is the upconversion emission of $NaYF_4$:Yb,Er (18,2%). The weaker light intensity after reaction indicates that the amount of nanoparticles is decreased.

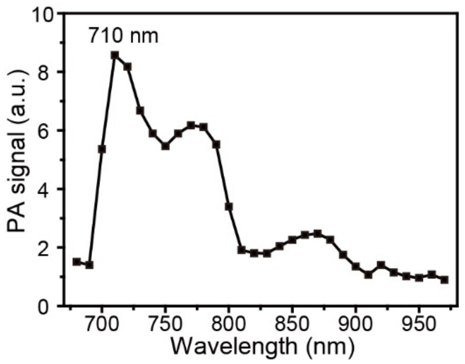

Figure S10. Photoacoustic signal of BPN at different excitation wavelengths.

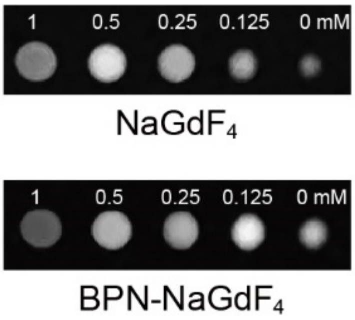

Figure S11. The magnetic resonance imaging of ligand-free $NaGdF_4$ and BPN-$NaGdF_4$ nanocomposites.
